# Persistent Pain as an Early Indicator for Operative Carpal Tunnel Revision after Primary Release: A Retrospective Analysis of Recurrent and Persistent Carpal Tunnel Syndrome

**DOI:** 10.3390/healthcare11142100

**Published:** 2023-07-24

**Authors:** Daniel Georg Gmainer, Andrzej Hecker, Petra Brinskelle, Alexander Draschl, Patrick Reinbacher, Lars-Peter Kamolz, David Benjamin Lumenta

**Affiliations:** 1 Division of Plastic, Aesthetic and Reconstructive Surgery, Department of Surgery, Medical University of Graz, 8036 Graz, Austria; danielgmainer@gmx.at (D.G.G.); david.lumenta@medunigraz.at (D.B.L.); 2 Research Unit for Digital Surgery, Department of Surgery, Medical University of Graz, 8036 Graz, Austria; 3 COREMED-Centre for Regenerative Medicine and Precision Medicine, Joanneum Research Forschungsgesellschaft mbH, 8010 Graz, Austria; 4 Department of Orthopaedics & Traumatology, Medical University of Graz, 8036 Graz, Austria

**Keywords:** carpal tunnel syndrome, pain, persistence, recurrence, revision carpal tunnel release, revision surgery, secondary carpal tunnel surgery

## Abstract

Background: Prolonged symptoms of carpal tunnel syndrome (CTS) after primary carpal tunnel release (CTR) can reduce the quality of life and lead to multiple referrals across specialties. The following study aimed to identify differences in symptoms, signs, and intraoperative findings between recurrent and persistent CTS cases to avoid undesired outcomes after primary CTR. Methods: A retrospective analysis was conducted on revision CTRs performed between 2005 and 2015 using literature-based definitions for recurrent (a relapse of symptoms occurs following a symptom-free period of ≥3 months) and persistent (symptoms persisting longer than three months after surgical release) CTS. The parameters assessed were symptoms, clinical signs, and intraoperative findings. Results: Out of 53 cases, 85% (n = 45) were external referrals, whereby our own revision rate was 0.67% (n = 8/1199). Paresthesia and numbness were frequent in both groups; however, abnormal postoperative pain was reported more often in persistent cases (86%; n = 30/35) in comparison to recurrent cases (50%; n = 9/18; *p* = 0.009). Scarring around the median nerve was observed in almost all recurrent cases (94%; n = 17/18) and in 40% (n = 14/35) of persistent cases (*p* < 0.001). Incomplete division of the palmar ligament was the primary cause for revision in the persistent cohort (49%; n = 17/35). Conclusions: For patients experiencing symptoms for more than three months after CTR, primarily presenting as pain, it is advisable to consider referring the patient to a certified hand clinic for additional evaluation.

## 1. Introduction

Carpal tunnel syndrome (CTS) is the most common hand entrapment neuropathy accounting for 90% of all nerve compression syndromes [1,2]. Its prevalence in the general population is reported as 17.4% in females and 10.4% in males [3]. According to a 2019 report, an annual increase in diagnosed CTSs from 2% to 4% and an increase in release surgeries from 5% to 6% was found in the Swedish population [4]. The primary cause of CTS is a volume mismatch within the carpal tunnel and its associated structures due to (external) pressure, ischemia, repetitive strain, as well as anatomical factors affecting median nerve movement in the carpal tunnel. Various predisposing factors such as genetics, pregnancy, diabetes, thyroid disorders, rheumatoid diseases, or kidney diseases can increase the chance for this phenomenon [5,6,7]. Risk factors associated with a higher revision rate include endoscopic carpal tunnel release, bilateral carpal tunnel release, male sex, smoking, rheumatoid arthritis, and bilateral carpal tunnel release [8,9].

Despite the effectiveness of surgical carpal tunnel release (CTR) in resolving symptoms, a subset of patients (with an incidence ranging from 1% to 32%) continue to experience persistent or recurrent symptoms after the release being performed [3,10,11,12,13,14]. While various studies report revision rates ranging from 0.3% to 12% [14,15], larger series typically indicate a rate of around 5% [3,10]. Hence, given the high prevalence of CTS, even a small proportion of patients requiring revision surgery after primary release can result in a significant number of additional surgical interventions.

This investigation was conducted in response to an increase in external referrals for operative CTS revision in our department. An additional motivation was the recognition of several authors [16,17,18] regarding the importance of classifying symptoms and time intervals to ascertain the underlying causes of persistent and recurrent complaints in CTS revision cases. Therefore, a literature-based definition was used to distinguish between recurrent and persistent CTSs after primary CTR, whereas recurrent CTS is defined as symptoms recurring after a symptom-free interval of at least three months [19,20,21,22,23]. In this retrospective study, all operative revisions were grouped and analyzed according to the mentioned definition.

The current study aimed to determine differences in the distribution of symptoms, clinical signs, and intraoperative findings between recurrent and persistent CTS revision cases in order to avoid undesired outcomes after initial carpal tunnel release and improve the surgical treatment of CTSs.

## 2. Materials and Methods

Following institutional review board approval (29-194 ex 16/17), we conducted a search of the electronic patient records (EPR) in the institutional database using the ICD-10 code G56.0 for “carpal tunnel syndrome” to identify patients who had undergone revision CTR in the time period from 2005 to 2015. We manually retrieved and reviewed data from operative reports, outpatient notes, and pre-operative anesthetic records. Diagnostic data and operative reports from external referrals were unavailable for review.

All patients who underwent CTS revision surgery were included in the study. Exclusion criteria were defined as pregnancy, cervical spine diseases, or conditions with median nerve compression other than carpal tunnel syndrome. Data on various parameters such as sex, body mass index (BMI), pre-existing comorbidities, clinical signs, electrophysiological diagnostic tests (nerve conduction velocity (ncv)), imaging studies, time-to-revision, the number of previous external revisions, intraoperative findings, and postoperative recovery were obtained (Table 1, Table 2 and Table 3). Time till recurrence refers to the time interval from initial release to the date of recurrent or persistent symptom description in days. Postoperative recovery was defined as the complete freedom of symptoms or a substantial improvement with only mild residual symptoms throughout the clinical follow-up period. Additionally, a web-based randomizer (https://www.zufallsgenerator.net/wuerfel-zufallsgenerator.html (accessed on 25 November 2015)) was used to select one hand in patients with bilateral CTS.

In our clinical routine, all the patients underwent examination and electrophysiological studies, if not already available, before revision surgery. Ultrasound studies were conducted when electrophysiological studies were inconclusive or requested by the attending surgeon in charge. All operative revisions were performed by board-certified hand surgeons using an open surgical approach with appropriate proximal and distal lengthening of the initial incision resulting from the primary release. Routine follow-up checks were arranged 2, 14, and 90 days after surgery.

We distinguished recurrent from persistent CTS based on a symptom-free interval of at least three months, as stated in the literature, and grouped accordingly [19,20,21,22]. The main diagnostic criteria commonly recorded at our institution were pain, numbness in the median nerve sensory distribution, nocturnal numbness, weakness and atrophy of the thenar muscles, Tinel’s sign, Phalen’s sign, and the loss of two-point discrimination [24].

The collected data were transferred to a spreadsheet program (Microsoft^®^ Excel^®^, Redmond, WA, USA) and statistical analyses were performed with IBM^®^ SPSS^®^ (Statistics 24, Armonk, North Castle, NY, USA). The Kolmogorov–Smirnov test was used to analyze data with a parametric distribution, while continuous data were analyzed using two-tailed *t*-tests or non-parametric Mann–Whitney U tests. Fisher’s exact test was used for categorical data analysis in intergroup comparisons. The statistical analysis involved calculating the means and standard deviations (SD) of continuous variables and frequencies and relative frequencies for categorical variables. For non-normal distributed data, the median and interquartile range (IQR) were used. A two-tailed *p*-value less than 0.05 was set as the threshold for statistical significance.

## 3. Results

### 3.1. Study Enrollment

**Figure 1:**
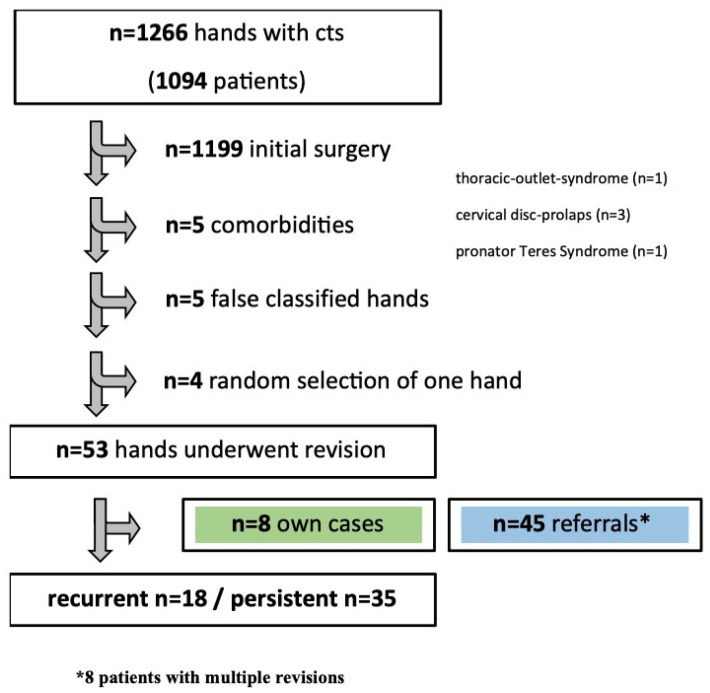
Study enrollment. Flowchart of inclusion and exclusion of patients who underwent revision carpal tunnel release (CTR).

The present study involved a retrospective analysis of 1266 hands diagnosed with CTS in 1094 patients, retrieved through a search of the institutional ERP system. Of those, 1199 were initial CTRs, and 67 were revision surgeries. Five cases were excluded due to cervical spine disc prolapses (n = 3), thoracic outlet syndrome (n = 1), and pronator teres syndrome (n = 1). Additionally, five hands were incorrectly coded and four patients had bilateral hand involvement, resulting in 53 hands being analyzed (Figure 1).

Fifty-three revision CTR cases were performed in our division during the study period. Eight patients underwent revision CTR at our department, representing 15% of the cases. The remaining 45 cases were external referrals for revision surgery from non-hand centers. The 53 cases were further divided into two groups for analysis: 18 recurrent and 35 persistent CTS cases (Figure 1).

Seven patients were referred to us due to persistent complaints in the same hand and had undergone two previous surgeries on the affected hand elsewhere. In the recurrent group, one patient had previously undergone three surgeries on the affected hand, with the most recent procedure being performed two years before being referred to our department. Among our own revision cases (n = 8), three of them were recurrent and five of them were persistent.

### 3.2. Patient Characteristics, Comorbidities, Time Till Revision Surgery, and Follow-Up

Descriptive patient characteristics and comorbidities are presented in Table 1. Sex, age, body mass index (BMI), smoking status, and comorbidities were evenly distributed, with no significant differences between the two groups. Time until CTS revision and end of follow-up, however, were not normally distributed. Recurrent cases had a significantly longer median duration from initial CTR until revision surgery compared to the persistent group (121.0 months (IQR: 103) vs. 7.0 (IQR: 47) months, *p* < 0.001) (Table 1). The median duration from revision surgery to the end of the follow-up period was 1.03 (IQR: 3) months and 1.0 (IQR: 68) month in the recurrent and persistent groups, respectively (Table 1).

### 3.3. Time until Recurrence

The recurrent cases (n = 18) were categorized based on the time until recurrence. One patient (5.6%) experienced recurrent symptoms between 3 and 6 months postoperatively, while no case had recurring symptoms between 6 months and 1 year after surgery. Eleven patients (61.1%) reported recurrent symptoms between 1 and 10 years, and six patients (33.3%) developed recurrent symptoms after more than 10 years.

### 3.4. Revision Surgery and Intraoperative Findings

Among the recurrence group, 17 individuals (94%) demonstrated scarring or fibrosis around the median nerve as the primary intraoperative finding. In contrast, the incomplete division of the transverse carpal ligament or antebrachial fascia was responsible for persistent complaints in 19 cases (54%). Of the persistent cases, 14 patients (40%) had scarring, among them one neuroma (3%) and one fibroma (3%) were identified. Notably, scarring was identified as the main finding in recurrent CTS patients (94%), and the difference was statistically significant (*p* < 0.001), as seen in Table 2.

The main intraoperative finding in our own recurrent cases was scarring in two patients and incomplete division in one case. Among the five persistent cases, two were attributed to incomplete division, another two to scarring, and one to fibroma formation.

### 3.5. Postoperative Improvement

Twelve (67%) of the recurrent cases achieved postoperative improvement, while four (22%) were lost to follow-up. In the persistent group, 25 patients (72%) achieved satisfying postoperative recovery, while five (14%) individuals were lost to follow-up. Of our own revision cases, all recurrent patients and four patients in the persistent group achieved full recovery without residual symptoms (Table 2).

### 3.6. Symptoms and Clinical Signs

When comparing the symptoms and clinical signs of the persistent and recurrent groups, pain in the median nerve distribution was found to be significantly more frequent in the persistent group (*p* = 0.009; Table 3). Specifically, 86% of patients with persistent CTS claimed to suffer from pain, whereas only 50% of patients with recurrent CTS reported the same. However, there were no statistically significant differences between the two groups concerning other symptoms or clinical signs (Table 3).

## 4. Discussion

In this retrospective comparative analysis, we investigated potential differences in symptom distribution, clinical signs, and intraoperative findings between recurrent and persistent CTS cases following CTR. Our study showed that the temporary increase in revision rates was primarily attributed to patients who were referred from external sources and had undergone multiple releases before being referred to our institution, a certified hand center. Compared to the revision rates in the literature, ranging from 0.3 to 12% [15,16], our own revision rate of 0.67% (n = 8/1199 cases) was at the lower end of the spectrum. After revision surgery, our case series demonstrated an overall improvement in 84.1% of the patients, excluding those lost to follow-up (n = 9), irrespective of the type of referral. Other authors with a comparable number of cases achieved improvement rates of 68.4% [25] and 82% [26].

Several studies have noted that iatrogenic injuries, incomplete CTR, and scar formation are the primary causes of unresolved symptoms in CTS patients after initial release [17,20,27,28]. Tung and Mackinnon [17] have categorized symptoms into persistent, recurrent, and new based on pre- and postoperative complaints and a symptom-free interval to identify patients requiring surgical re-intervention after primary CTR. Their findings revealed that incomplete release, the constriction of the antebrachial fascia, and misdiagnosis contribute to persistent CTS. However, recurrent symptoms usually occur due to pathological scar formation or scarring with subsequent healing of the transverse carpal ligament. The appearance of new or different symptoms than reported before initial CTR might indicate iatrogenic injury [17]. Similar classification approaches have been adopted by other authors [18,26,27,29]. Furthermore, Stütz et al. [20] found that symptoms that reappear after initial pain relief are mainly associated with the formation and solidification of scars, which usually takes around three to six months. Given the mentioned duration of scar and solidification formation, various studies, including the current investigation, have used a three-month symptom-free interval to distinguish between recurrent and persistent cases [17,21,22,30]. However, as most recurrent CTS cases (61.1%) in our study population reported recurrent symptoms 1 to 10 years after surgery, our findings suggest that scar formation significantly contributes to unresolved symptoms in CTS patients even years after the initial release.

Except for pain, all the other assessed symptoms and clinical signs were equally distributed between our two groups. The most important finding of our study was the identification of significantly higher levels of pain in patients with persistent CTS prior to undergoing revision CTR, compared to those with recurrent symptoms. Specifically, 86% of the persistent CTS cases reported pain exceeding expected postoperative levels and duration, compared to 50% in the recurrent group (*p* = 0.009). Our finding is consistent with a study conducted by Zieske et al. [31], which reported that patients suffering from persistent symptoms are more likely to experience pain than those with recurrent CTS (83% vs. 53%, respectively). We attribute the higher levels of pain in our persistent CTS group mainly to a combination of incomplete release and scar formation around the median nerve, which were the main intraoperative findings. Excessive fibrous tissue around the median nerve within the carpal tunnel can result in scar fixation of the median nerve under the flexor retinaculum, subsequently leading to painful traction neuropathy [32]. However, a prior study evaluating the predictive value of symptom duration on the outcome after initial CTR did not show an independent association with worse outcomes [33].

Masud et al. [2] conducted a study investigating the influence of preoperative duration and severity of CTS symptoms on the outcome of CTR using an open approach. According to their findings, patients with a median preoperative pain duration of less than 12 months experienced relief within 4 weeks after CTR, whereas those with pain persisting more than 12 months required 12 weeks for pain relief after surgery. Similarly, Eisenhardt et al. [34] reported an extended recovery period of 25 days in patients who had experienced symptoms of CTS for more than 12 months compared to 16 days in patients with less than a 12-month duration of symptoms before performing endoscopic CTR. Both studies highlight that a prolonged symptom presentation can lead to an extended recovery period regarding pain. It is worth noting that both studies indicate that the average recovery period for pain after CTR does not exceed 12 weeks [2,34]. Beyond this time frame, improvements in pain seem highly unlikely. Although a previous study did not demonstrate an independent relationship between symptom duration and worse outcomes after initial CTR [32], we believe that the presence of persistent pain lasting longer than three months without the tendency of pain reduction following CTR should prompt early reassessment and consideration for timely intervention through revision CTR. This proactive approach could be crucial to prevent potential long-term median nerve damage and increase the quality of life.

Persistent pain can therefore be used as an early clinical indicator for the need of a subsequent revision CTR. However, it should be differentiated from local pain in the scar area, which can be provoked by light pressure or touch and usually resolves spontaneously. Moreover, it should be kept in mind that pain is perceived subjectively; thus, potential nerve damage may also vary and lead to different prognoses. Furthermore, it is essential to distinguish between pain due to median nerve entrapment and other conditions that can cause similar symptoms in the hand and wrist area. Pillar pain, which is characterized by pain in the thenar and hypothenar eminences with possible weakness of pinch and grip strength [35], and piso-triquetral pain syndrome, which causes pain at the base of the hypothenar area due to changes in forces over the piso-triquetral joint [36], are examples of such conditions. Additionally, patients with complex regional pain syndrome (CRPS) experience neuropathic pain, cold sensitivity, variable swelling, and difficulty sleeping, typically occurring 1–3 weeks after CTR [37]. Clinical hints of CRPS should prompt an early referral to a pain specialist. Therefore, distinguishing between persistent pain after CTR and other types of pain is crucial to determine the appropriate course of treatment.

While pathological scar formation was identified as the main revision finding in our recurrent cases (94%), incomplete division of the transverse carpal ligament or antebrachial fascia was the leading discovery in persistent cases (54%). This aligns with the findings of other studies, which attributed incomplete release to persistent symptoms and circumferential fibrosis around the median nerve to recurrent cases [26,38]. Moreover, increased scar formation is associated with prolonged immobilization, poor hemostasis, hematoma formation, or inappropriate hand therapy [17,38]. The negative impact of restrictions in postoperative care on early rehabilitation has also been reported in previous studies [39,40]. However, one persistent case in the current study was caused by neuroma formation resulting from iatrogenic injury, therefore requiring median nerve reconstruction of 70% of its cross-sectional area over a four centimeter section. While the presentation of new symptoms seemed to be very likely, we were not able to confirm those from the documentation in the medical reports.

Stütz et al. [20] identified incomplete division of the transverse carpal ligament in 108 of 200 cases. In their study, 93 of 108 patients (86%) reported no relief and unchanged symptoms after initial CTR, and in 15 cases, symptoms reappeared shortly afterward. These and other preventable causes amount to 83% (166 of 200) of avoidable revision surgeries [20]. In our patients, preventable revision surgery causes, including incomplete divisions and one neuroma, were responsible for 21 cases (40%). This highlights the importance of not treating CTR as a simple procedure. Instead, it underscores the necessity for meticulous surgical interventions, even for this “routine surgery”, and the requisite expertise to perform it. Furthermore, partial resection of the transverse carpal ligament, instead of just splitting it during endoscopic release, is recommended due to evidence supporting its ability to reduce the risk of rejoining and therefore resulting in a decreased revision rate [41].

Our study population’s sex distribution is consistent with the reported prevalence of CTS and previous studies on revision cases that have indicated a higher incidence among females. The sex distribution of our study population aligns with the reported prevalence of CTS and with previous studies on revision cases, which have shown a higher incidence among females [20,30]. As smoking has been linked to a higher rate of revision CTR [8] and being overweight has been identified as a dose-dependent risk factor in CTS development [42,43], with an increased BMI being associated with a larger median nerve cross-sectional area [44], we aimed to explore potential differences in these factors between recurrent and persistent cases. Despite no significant difference regarding the BMI between the recurrent and persistent group, the fact that both groups had mean BMIs classified as obese aligns with the already mentioned higher risk of CTS development in the overweight population. Regarding the smoking status, we found a higher proportion of smokers in the recurrent group (50%, n = 9) in comparison to the persistent group (31%, n = 11). These findings are consistent with those of Zieske et al. [30], who also reported a higher proportion of smokers in the recurrent group (53%, n = 10) compared to the persistent group (31%, n = 13). However, a recent meta-analysis of several case-control and cohort studies found no association between ever, past, or current smoking and CTS formation [45].

The present study has several limitations, including its retrospective design, a relatively small sample size consisting mostly of patients who underwent primary CTR in other institutions, variations in documentation styles, and the fact that all parameters were evaluated by multiple board-certified hand surgeons, which made data acquisition and comparability challenging. Despite several attempts to exclude postoperative pain around the scar area which can be triggered by palpation, analyzing the quality of excessive pain in the distribution of the median nerve after CTR in more detail, such as using the VAS (visual analogue scale), was not possible. It should also be noted that symptoms, especially pain, are perceived subjectively, potentially resulting in a recall bias regarding the assessment of CTS symptoms. Scarring was documented in most surgical reports. However, it was not always clear whether it was the cause of persistent or recurrent symptoms or simply a result of previous surgeries, making the intraoperative determination of the “cause” of recurrent or persistent CTS a subjective matter and far from definitive; thus, the cautious interpretation of the results. Here, future prospective studies should quantitatively evaluate fibrosis and scarring intraoperatively. Furthermore, the comparison of symptoms before primary CTR with postoperative symptoms was not possible due to a lack of documentation or incomplete external reports from referrals. Additionally, incomplete division of the carpal ligament was considered as a primary revision finding only if explicitly stated in the operative report. Furthermore, it is important to note that our study had a limited follow-up period, which restricts our ability to assess the long-term outcomes of revision CTS in our study population. Future studies with extended follow-up durations are needed to understand the long-term effects more comprehensively.

## 5. Conclusions

This study identified that patients with persistent CTS experienced considerably higher pain levels before revision CTR than those with recurrent symptoms. Since improvements in persistent cases are unlikely beyond three months, for patients experiencing symptoms for more than three months after CTR, primarily presenting as pain, it is advisable to consider referring the patient to a certified hand clinic for additional evaluation.

## Figures and Tables

**Table 1:** Patient characteristics, comorbidities, time till revision surgery, and postoperative follow-up.

		Recurrent (n = 18)	Persistent (n = 35)	*p*-Value
sex (female:male)	n (%)	12:6 (67%:33%)	21:14 (60%:40%)	0.768
age [years]	mean (±SD)	55.9 (±11.8)	62.6 (±12.4)	0.065
BMI	mean (±SD)	29.4 (±6.0)	28.7 (±5.8)	0.679
smoking	n (%)	9 (50%)	11 (31%)	0.237
diabetes	n (%)	4 (22%)	7 (20%)	1.000
kidney disease	n (%)	0 (0%)	3 (9%)	0.543
endocrinological disorders	n (%)	6 (33%)	15 (43%)	0.565
rheumatological disorders	n (%)	6 (33%)	17 (49%)	0.384
time until CTS revision surgery [months]	mean (±SD) median (IQR)	106.2 (±61.0) 121.0 (103)	9.9 (±11.2) 7.0 (47)	<0.001 <0.001
end of follow-up [months]	mean (±SD) median (IQR)	6.6 (±21.4) 1.0 (3)	5.2 (±13.3) 1.0 (68)	0.733 0.711

SD: standard deviation; IQR: interquartile range.

**Table 2:** Detailed revision surgery data, intraoperative findings, and frequency of sufficient recovery after revision surgery.

		Recurrent (n = 18)	Persistent (n = 35)	*p*-Value
duration of surgery [min]	mean (±SD) median (IQR)	60.8 (±38.4) 44.5 (51.5)	57.8 (±46.3) 48.0 (46.0)	0.812 0.501
intraoperative findings				
scarring/fibrosis	n (%)	17 (94%)	14 (40%)	<0.001
incomplete division	n (%)	1 (6%)	17 (49%)	0.002
tight antebrachial fascia	n (%)	0 (0%)	2 (6%)	0.543
neuroma	n (%)	0 (0%)	1 (3%)	1.000
fibroma	n (%)	0 (0%)	1 (3%)	1.000
sufficient recovery	n (%)	12 (67%)	25 (72%)	1.000

SD: standard deviation; IQR: interquartile range.

**Table 3:** Symptoms and clinical signs prior to revision surgery.

		Recurrent (n = 18)	Persistent (n = 35)	*p*-Value
pain	n (%)	9 (50%)	30 (86%)	0.009
paresthesia	n (%)	13 (72%)	27 (77%)	0.743
numbness	n (%)	5 (28%)	11 (31%)	1.000
weakness	n (%)	3 (17%)	9 (26%)	0.730
Tinel’s sign	n (%)	11 (61%)	19 (54%)	0.772
Phalen’s sign	n (%)	8 (44%)	10 (29%)	0.359
(hypo-)thenar atrophy	n (%)	4 (22%)	11 (31%)	0.539

## Data Availability

The datasets generated and/or analyzed during the current study are available from the corresponding author upon reasonable request.
